# Manipulation of dangling bonds of interfacial states coupled in GeTe-rich GeTe/Sb_2_Te_3_ superlattices

**DOI:** 10.1038/s41598-017-17671-w

**Published:** 2017-12-11

**Authors:** Zhe Yang, Ming Xu, Xiaomin Cheng, Hao Tong, Xiangshui Miao

**Affiliations:** 1 Wuhan National Laboratory for Optoelectronics, Huazhong University of Science and Technology, Wuhan, 430074 China; 2 School of Optical and Electronic Information, Huazhong University of Science and Technology, Wuhan, 430074 China; 3 Wuhan National High Magnetic Field Centre, Huazhong University of Science and Technology, Wuhan, 430074 China

## Abstract

Superlattices consisting of stacked nano-sized GeTe and Sb_2_Te_3_ blocks have attracted considerable attention owing to their potential for an efficient non-melting switching mechanism, associated with complex bonding between blocks. Here, we propose possible atomic models for the superlattices, characterized by different interfacial bonding types. Based on interplanar distances extracted from ab initio calculations and electron diffraction measurements, we reveal possible intercalation of dangling bonds as the GeTe content in the superlattice increases. The dangling bonds were further confirmed by X-ray photoelectron spectroscopy, anisotropic temperature dependent resistivity measurements down to 2 K and magnetotransport analysis. Changes of partially coherent decoupled topological surfaces states upon dangling bonds varying contributed to the switching mechanism. Furthermore, the topological surface states controlled by changing the bonding between stacking blocks may be optimized for multi-functional applications.

## Introduction

The design of heterostructures and superlattices containing artificial interfaces has received considerable attention owing to the intriguing physical properties and functionalities of these materials, including their topological behaviour and superconducting properties^1,2^. Many of these interesting phenomena are attributed to atomic rearrangements in the vicinity of superlattice interfaces^3^. Recently, interfaces between phase-change materials have been constructed by textured growth, based on GeTe-Sb_2_Te_3_ superlattices. These systems show potential for greatly reduced power consumption in phase-change memory devices^4^. As an alternative to conventional melting-quench phase-change processes, several non-melting switching mechanisms, including vertical displacement of atomic layers and strain engineering, have been proposed^4–10^. However, these switching mechanisms have not been widely investigated because the ground state of GeTe/Sb_2_Te_3_ superlattices remains a subject of debate owing to the incompatible bonding types at their interfaces^11–17^. Experimental observations have suggested that interfacial 2D Te-Te bonding occurs. This is a type of van der Waals (vdW) bonding, which contributes to the formation of various trigonal Ge-Sb-Te blocks at interfaces^15–17^. However, the dangling bonds of nano-sized GeTe blocks cannot be neglected and have played more prominent roles in superlattices than in classic GeTe or Ge-Sb-Te films^18,19^. For example, the intercalation of nano-sized GeTe blocks with varying thickness can allow the topological surface states in superlattices to be manipulated depending on changes of the interactions between two blocks^20–22^. In studies of the local structure of GeTe films, the chemical bonds have been shown to gradually change with decreasing film thickness to nano scale^23,24^. Thus, an unknown relationship exists between the incompatible bonds (reconfiguration of the 2D and 3D dangling bonds) and interfacial states in superlattices. As joule heating contributes to the structure relaxation, annealing may also be another effective method to explore such relationship and provide information about the configuration of the superlattices. Furthermore, this understanding might conversely guide the design of attractive functionalities based on the nontrivial interfacial states and electron-spin interactions of various GeTe/Sb_2_Te_3_ superlattices^25–28^.

To date, most investigations into ground-state atomic configurations have concentrated on interfacial phase-change memory (iPCM) superlattices^4^, consisting of ultra-thin GeTe blocks (<1 nm). However, in these samples, the GeTe blocks tend to completely intermix with Sb_2_Te_3_ blocks upon annealing, resulting in the disappearance of the interfaces^15–17^. What’s more, topological surface modes can also hybridize across GeTe blocks besides of Sb_2_Te_3_ with thin thickness^20,29^, resulting in the coupling or break down of the topological surfaces states. These issues may be addressed by increasing the GeTe content of the superlattices to maintain the interfaces. Furthermore, a well-established low-temperature transport measurements in nanostructured systems^30–32^ are effective for obtaining more information about the interfaces.

To this end, we first fabricated several GeTe-rich superlattices samples with different annealing temperatures. Three theoretical models were used to predict the properties of the GeTe-rich superlattices, with distinct chemical bonds at their interfaces, by ab initio calculations. The layered models were then confirmed by a series of experiments, including the comparison of interplanar distance deduced separately from electron diffraction experiments and DFT calculations, X-ray photoelectron spectra (XPS), qualitatively different behaviour of the in-plane (ρ_ab_) and out-of-plane (ρ_c_) resistivity as well as the detection of partially decoupled topological surfaces. Our results demonstrate a relationship between the chemical bonds and the topological surface states. The formation of 2D Van der Waals (vdW) interfacial bonds was disfavoured under Joule heating of the GeTe-rich sample owing to energetic considerations. These results indicated the presence of dangling bonds and how they change the topological surface states coupling. Together our findings contribute to an in-depth understanding of GeTe-rich superlattice configurations and the interfacial states associated with their switching mechanism.

## Results and Discussion

In general, iPCM superlattices are described as [GT_n_/ST_m_]_T_, where GT and ST denote GeTe and Sb_2_Te_3_ blocks and the subscripts n and m indicate the thickness (nm) of the blocks. The annealing temperature is denoted by the subscript T. More details of the sample preparation are given in refs^33,34^. In our experiments, we inserted thick GeTe blocks to avoid dissolution of the superlattice structure owing to atomic intermixing or hybridization. The interfacial states were designed to be enhanced by decreasing the thickness of the Sb_2_Te_3_ block to approximately 2QL (~2 nm) and increasing the superlattice period by 50 times to reach a high surface-to-bulk ratio. The thickness of each block was controlled by the sputtering time and measured with a scanning transmission electron microscope (STEM). The crystallization temperature of Sb_2_Te_3_ (<100 °C) is low; hence the GeTe was grown on a Sb_2_Te_3_ substrate along the [111] direction which has a small lattice mismatch^35^. The as-deposited Sb_2_Te_3_ was crystalline with distinct layered properties, as shown in Figure S1 (a) (supplementary material). Because the crystallization temperature of GeTe is approximately 230 °C, the annealing temperatures for the two samples [GT_4_/ST_2_]_250 °C_ and [GT_4_/ST_2_]_300 °C_ were 250 and 300 °C, respectively, leading to different interfacial characteristics. Another two reference samples of GeTe and Sb_2_Te_3_ were prepared and annealed at the same temperatures for comparison with the superlattice materials (See more in supplementary).

### GeTe-rich superlattice structure model

**Figure 1:**
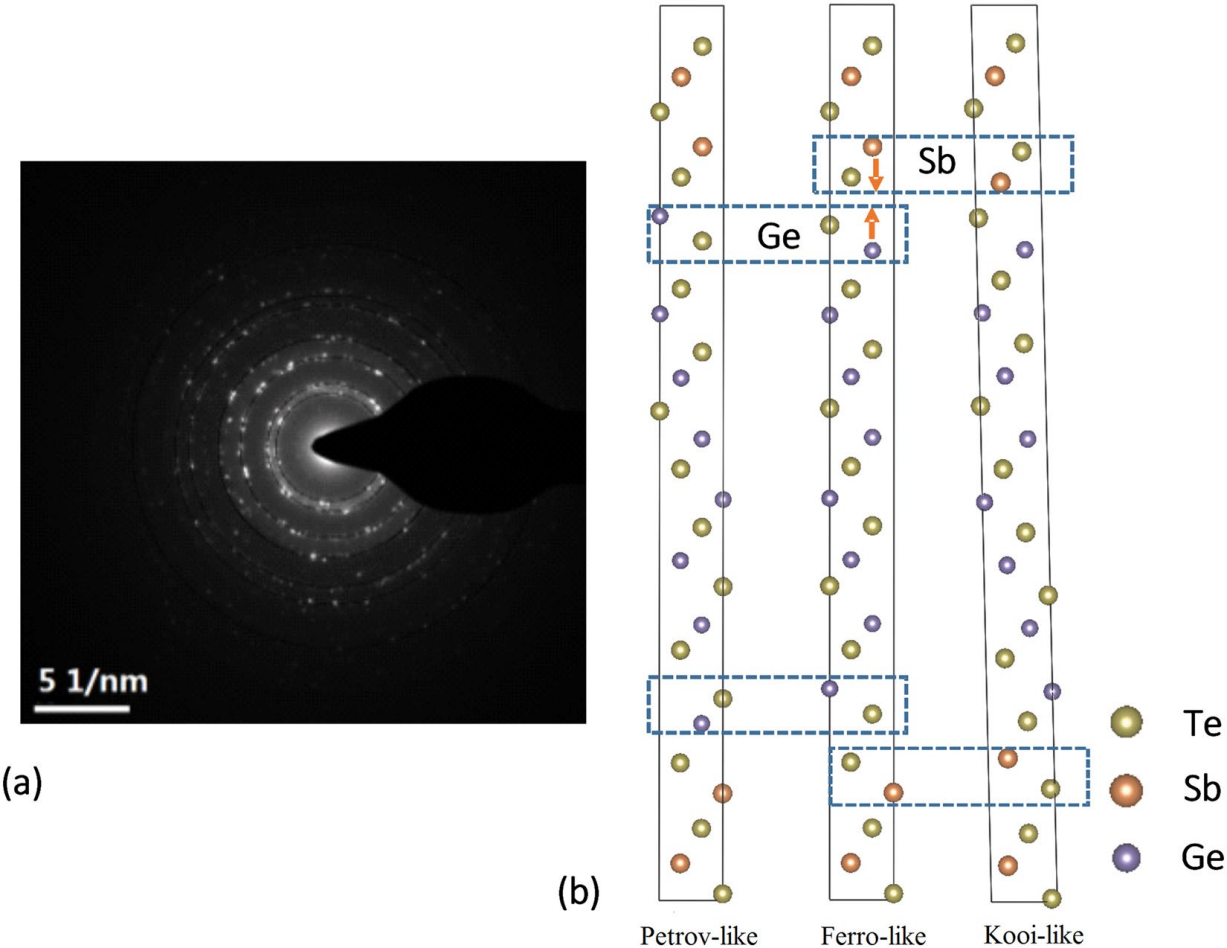
(**a**) Electron diffraction pattern of the crystalline superlattice [GT_4_/ST_2_]_250 °C_. (**b**) Models of GeTe-rich GeTe/Sb_2_Te_3_ superlattice. Atomic stacking sequences (Ge-Te layers and Sb-Te layers) were assumed to be switched near the interfaces between GeTe and Sb_2_Te_3_ compared with those of the Ferro-like Model.

There are many possible structure configurations at the interfaces with various atomic arrangements, especially considering intermixing in GeTe/Sb_2_Te_3_ superlattice. However, not all structure configurations are reasonable according to structure energy, stability, chemical bonds and so on. Due to the complexity of this issue, we need to refine and simplify the models for theoretical calculations referred to massive previous work^5–17^. In general, there are four possible stacking sequences for the GeTe/Sb_2_Te_3_ SLs which have been widely accepted and investigated, namely, the Petrov (P) [–Te-Ge-Te-Sb-Te-Sb-Te-Ge-Te–], the Kooi (K) [–Te-Sb-Te-Ge-Te-Ge-Te-Sb-Te–], the inverted Petrov (iP) [Ge-Te–Te-Sb-Te-Sb-T–Te-Ge] as well as the Ferro (electric) (F) phase [Te-Sb-Te-Sb-Te–Te-Ge-Te-Ge]^5,6,10–14^. The notion “-” sign denotes covalent bonds and the “–” sign denotes vdW interaction. In the earlier Transmission electron microscopy (TEM) and theoretical calculation work, real structures are likely to be the mixture of these four prototype models^10,15^. Thus, the complex structural configuration puzzle is supposed to be resolved by making use of these four sequences. Based on this, we take the increasing GeTe portion property into consideration and propose our models correspondingly. They are Ferro-like, Petrov-like and Kooi-like models respectively shown in Fig. 1. Different from the prototype models, these three models feature a vacancy sublayer at Ge sites and don’t obey the strict stoichiometry for the thick GeTe block. This is consistent with the real vacancies that exist in GeTe blocks of superlattices^11^ and in line with the high carrier densities measured in superlattices compared with that of the bulk stacking components (See supplementary).

Based on the structural stability, the calculated energy of the Ferro-like Model was least which suggested the higher possibility of this model to exist in the samples. However, the Ferro-like Model faces the incompatible bond issue that Sb_2_Te_3_ is a typical vdW-bonded material with chemically passive Te surfaces and doesn’t prefer to directly connect with the Te atom in 3D covalent bonded GeTe^15–17^. Thus, the active atomic layers are likely to change their sequence and become intermixed at junctions of the superlattice^16^, resulting in the possible existent of the Kooi-like and Petrov-like Models for GeTe-rich superlattices. It is noted that we don’t consider the iP-like phase in our models. That is because, on one hand, the energy of interface terminated by Ge atoms is much higher compared to that of the interface with Te termination^10^. On the other hand, the GeTe portion in our case is much more than 2 buckled bilayers (BLs), that is not suitable for the presence of stacking sequence [-Ge-Ge-]. To validate the configurations further, we compared the total energy of the other two models. As shown in the first line of Table 1, the calculated energies of the Petrov-like (Ge-Te switching) models were lower than that of the Kooi-like Model (Sb-switching), implying that Kooi-like Model is most unstable with GeTe rich property. Furthermore, we theoretically calculated the interplanar distances of the three models, as listed in Table 1. Comparing the three columns, the difference of the interplanar distance between the Kooi-like and Ferro-like Models was more obvious than that between Petrov-like and Ferro-like Models. Thus, compared with the Sb-switching in Kooi-like model, the Ge-switching process in Petrov-like model was more energetically favourable with less structural relaxation. Though these three proposed models are most reasonable candidates for the GeTe-rich GeTe/Sb_2_Te_3_ superlattices, they still need to be further distinguished by the following experiments.

**Table 1:** Comparison of interplanar distance between experimental ([GT_4_/ST_2_]_250°C_) and calculated results obtained from GeTe-rich GeTe/Sb_2_Te_3_ models.

Experiment (Å) (eV/atom)	Ferro-like (−4.030)	Petrov-like Model (−4.025)	Kooi-like Model (−4.024)
3.407	3.399	3.423	3.304
3.02	3.08	3.08	3.05
2.102	2.117	2.113	2.138
1.70	1.699	1.711	1.65
1.478	1.469	1.444	\
1.327	1.346	1.34	1.358
1.21	1.22	1.22	1.24

### Verification of layered GeTe-rich superlattice structure

To verify these three configurations in GeTe-rich samples, we firstly investigated crystalline superlattice specimens annealed at 250 °C by TEM imaging. We calculated the interplanar distances from electron diffraction patterns, which agreed well with the DFT results from Ferro-like and Petrov-like models with small structural relaxation. Considering the differences from the aspect of the chemical bond, there is no dangling bonds which are characterized by the unpaired electrons at the interfaces of the Kooi-like Model where the Ge-Te termination is passivated by a switched Sb-Te outmost layer. This model can be viewed as stacking of SbTe_2_ and Ge rich trigonal Ge-Sb-Te blocks connected by 2D vdW interfacial bonds. In contrast, in both Ferro-like and Petrove like Models, the dangling bonds are preserved due to the abrupt termination of Ge-Te, resulting in the unpaired electrons. Our electron diffraction results implied that 2D vdW bonding was not the only possibility for GeTe-rich superlattices. The Te dangling bonds might also be present at interfaces, as indicated by the Ferro-like and Petrov-like Models which were more consistent with the experimental value.

**Figure 2:**
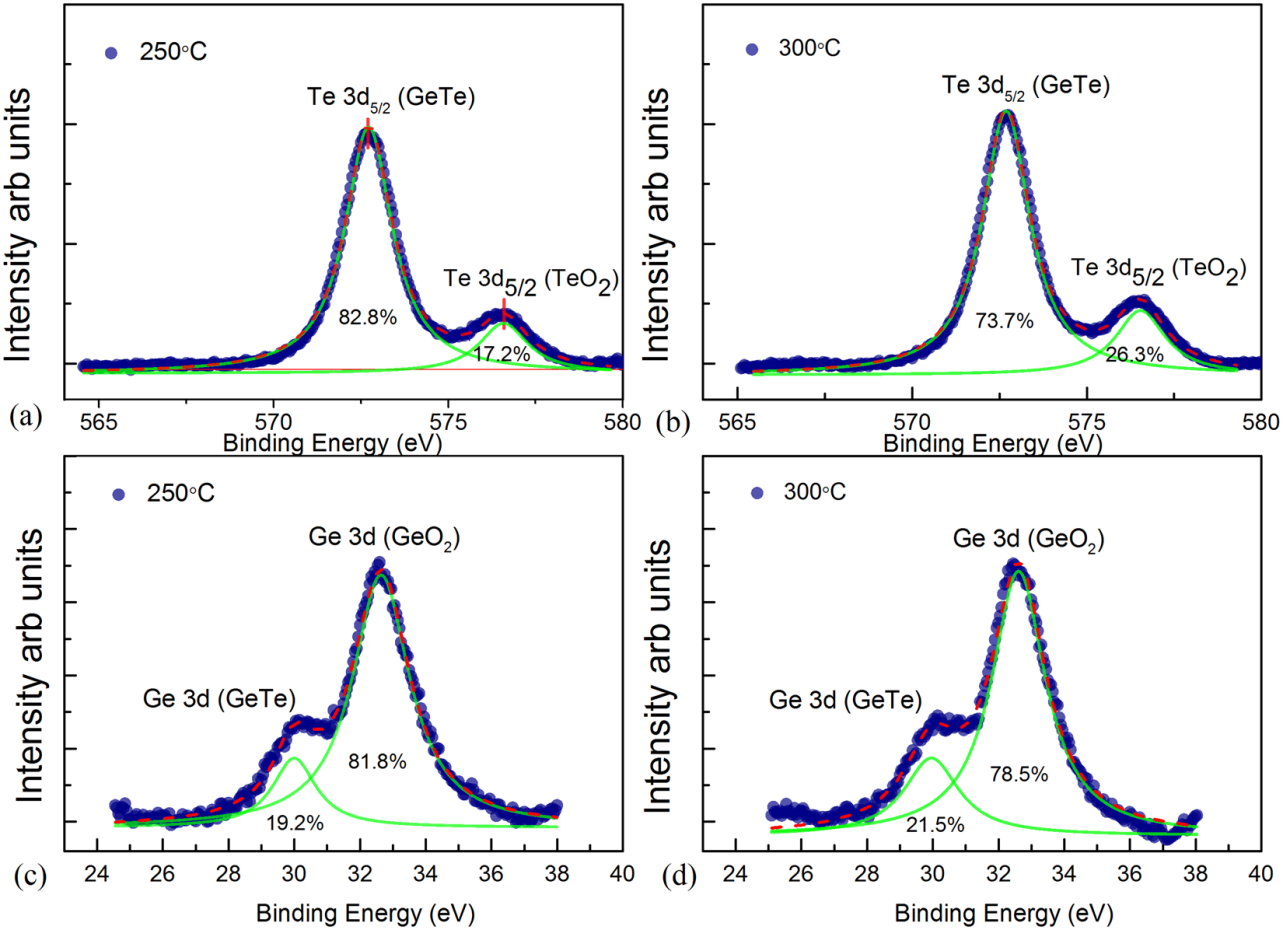
XPS measurement of GeTe rich GeTe/Sb_2_Te_3_ superlattices with different annealing condition. The green lines in the figure represent the peak fitting. (**a**) and (**b**) The Te 3d_5/2_ spectrums of superlattice samples annealed at 250 °C and 300 °C respectively. (**c**) and (**d**) The Ge 3d spectrum of superlattice samples annealed at 250 °C and 300 °C respectively.

In order to identify the Te dangling bonds further, the chemical state of GeTe-rich GeTe/Sb_2_Te_3_ superlattices were studied by XPS. As Te atoms in GeTe are difficult to oxidize, Te dangling bonds are considered as the main reason to form Te-O bonds because of the unpaired electrons^19^. Thus the more formation of Te-O bonds, the more existence of Te dangling bonds will be proven. Figure 2 shows the XPS spectra of Ge 3d and Te 3d_5/2_, respectively. The binding energy at 30.1 ± 0.03 eV and and 572.7 ± 0.06 eV are attributed to Ge 3d and Te 3d_5/2_ in GeTe, respectively, which is in agreement with values in the earlier work^36^. The peaks at 32.7 ± 0.04 eV and 576.5 ± 0.05 eV in Fig. 2 are due to GeO_2_ and TeO_2_ as reported before^36^. Comparing the Te spectrums in Figure (a) and (b), the relative value of peak area for the Te-O component was obviously larger in superlattice samples with increasing annealing temperature, suggesting the more dangling bonds before oxidation. In contrast, the relative of peak area for Ge-O changed slightly upon annealing. The Ge spectrums can confirm that the superlattice samples are fully oxidized and the oxidation process doesn’t affect by the annealing temperature. Thus the XPS study provided the evidence of the Te dangling bonds in superlattice and their variation upon annealing.

**Figure 3:**
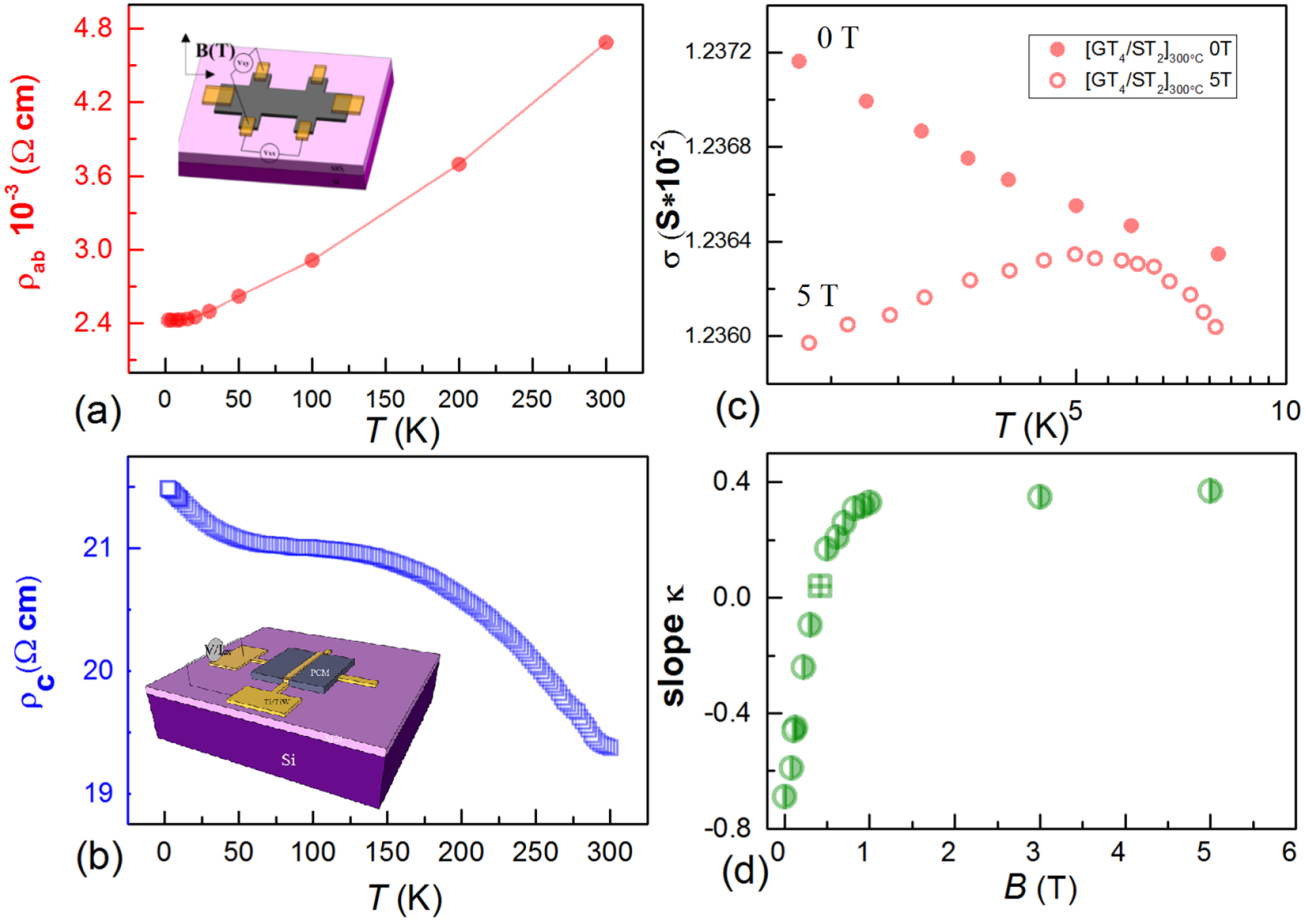
(**a**,**b**) Temperature dependence of the in-plane resistivity ρ_ab_ and out-of-plane resistivity ρ_c_ of [GT_4_/ST_2_]_300°C_. The insets show schematics of the Hall-bar structure used for in-plane transport (**a**), and cross-bar structure used for out-of-plane transport (**b**) measurements. (**c**) Conductance σ versus temperature for the [GT_4_/ST_2_]_300 °C_, and linear fitting of ln(T) at magnetic field strengths of 0 and 5 T, indicated by solid and hollow circles, respectively. (**d**) The slope of ln(T) fitting for [GT_4_/ST_2_]_300 °C_ as a function of the applied magnetic field, showing saturation at fields strengths greater than 1T.

To in-depth understand the above layered structure in terms of the motion of electrons in the superlattice, we conducted a systematic investigation of the temperature dependence of resistivity in the out-of-plane c-axis (ρ_c_) and in-plane (ρ_ab_) for a superlattice sample annealed at 300 °C. A Hall-bar structured device was designed for measurement of ρ_ab_ = (V_XX_/I_ex_) × (S_bc_/a). Here, V_XX_ is the longitudinal voltage, I_ex_ is the excitation current, S_bc_ is the cross-sectional area of Hall-bar and the parameter a is the length of Hall-bar device. Meanwhile, a crossbar structure was used for measurement of ρ_c_ = (V_c_/I_ex_ − R_TiW_ − R_ohm-c_) × (S_ab_/d) under the condition of ohmic contact. Here, V_c_ is the total voltage of the crossbar structure device with a small current excitation Iex, R_TiW_ is the resistance of electrodes, R_ohm-c_ is the ohmic contact resistance, S_ab_ is the area of contact between electrode and phase change superlattice, and d is the film thickness. Schematic diagrams of both structures are shown in Fig. 3(a) and (b) separately. More details about how to determine the relevant parameters can be found in the supplementary.

As expected for the layered structure, the transport was strongly anisotropic. As shown in Fig. 3(a) and (b), the magnitude of ρ_c_ was three orders of magnitude larger than that of ρ_ab_, and their temperature dependence was markedly different: ρ_ab_ appeared to be metallic-like while ρ_c_ was insulating. These behaviours suggest that the influence of the isotropic grain boundaries was negligible, as discussed in the supplementary information. Consulting other similar layered and anisotropic systems such as graphite^37^, we attribute this behaviour to the weakly interacting vdW gap (or Te-Te interaction), which intercepted electron conduction along the c-axis. Electrons are required to tunnel across the vdW gap or vacancy layer, leading to a much larger value of ρ_c_ than ρ_ab_, together with a very different temperature dependence^38^. This assumption is consistent with our proposed layered models and previously reported experimental observations of interfacial vdW bonding in iPCMs^15–17^. However, we still could not determine the interfacial structure as both interactions may hinder the motion of electrons across the blocks.

Different chemical bonds near interfaces are strongly related to the electronic structure through changes in the interlayer interaction^15^. So another approach to exploring the interfacial chemical bonds is to detect topological surfaces in GeTe/Sb_2_Te_3_ superlattice, which are controlled by interlayer interactions^20–22^. These phenomena can be detected by quantum transport at low temperature because of the co-contribution of weak antilocalization (WAL) and the electron-electron interaction (EEI) effects^39^. The WAL effect derives from a correction (Δσ) to electronic transport owing to the time-reversed closed trajectory of coherent electrons, which acquires a relative phase of π^40,41^. Because the topological surface and bulk states can both act as conducting channels and contribution to the WAL effect with different underlying mechanisms^41^, the existence of interfacial states, as well as the coupling strength, can be resolved from different WAL parameters deduced from the temperature dependence of Δσ and magnetoresistance^42,43^. The contribution of the WAL effect to the temperature dependence of Δσ can be quantitatively described by^43,44^:

1 $${{\rm{\Delta }}{\rm{\sigma }}}_{WAL-2D}=\frac{-\alpha {e}^{2}p}{2{\pi }^{2}\hslash }\,\mathrm{ln}\,\frac{T}{{T}_{0}}$$

Here, the subscript 2D indicates the equation is only valid for a two-dimensional system, T_0_ is the reference temperature, and p equals 1 for electron-electron inelastic scattering at low T. The prefactor α represents the coherently independent conducting channel number as each channel can contribute 0.5 to α^39,44^. As reported in many topological insulators or low dimensional systems with strong spin-orbit coupling^39–41^, both topological surface and bulk states can act as independent conducting channels and provide quantum correction Δσ in the equation 1. Meanwhile, when surface states get coherent coupled through bulk states, α will be smaller than 0.5 N (N is the number of surfaces states)^20,21,39,44^. In an all, though we could not determine the number of the surface states quantitatively, we could figure out the surface state and their trend of coherent coupling qualitatively by the comparison of α value to 0.5. It is commonly used in the exploration of topological insulators (TI) and exotic heterostructures^21,22^.

As seen in Fig. 3(c), the conductance σ showed a logarithmic T (lnT) dependence, decreasing at low temperature and zero magnetic field. When a magnetic field of 5 T was applied, the sign of dσ/dT changed from negative to positive below 4 K. This change can be explained by the semi-Boltzmann transport model corrected for 2D quantum interference^42^, written as σ = σ_0_ + Δσ_WAL_ + Δσ_EEI_, where σ_0_ is the Drude conductance, which saturates at low temperature^42^. The quantum correction Δσ_WAL_ arising from the WAL effect tends to be suppressed at low magnetic field and is described by Eq. (1). The quantum correction Δσ_EEI_ originates from electron-electron interactions (EEIs) and is unaffected by weak magnetic fields^43,45^. Unlike Δσ_WAL_, Δσ_EEI_ is negative and increases linearly with ln(T) for the 2D regime^43^. To quantitatively distinguish the two effects, we used the slope κ (with the units of e^2^/πh) extracted from linear fits in the temperature range 2–4 K with different magnetic field strengths to describe the Δσ magnitude. (See more fitting details in supplementary material). With the use of Eq. (1) and the magnitude of Δσ_WAL_, the parameter α was calculated to be around 1.09, indicating that at least two coherent partially decoupled 2D channels were present in our system. This 2D transport result is notable for superlattice systems because the structures were dominated by the GeTe-Sb_2_Te_3_ interface rather than bulk carriers. We could use the above induced interfacial state, as indicated by the α value, to figure out the atomic structure models.

To reveal more about the topological surface states, we conducted magnetoresistance measurements which are also featured contributions from the WAL effect. The effect of WAL on the magnetoresistance at low magnetic field strength is described by the simplified Hikami–Larkin–Nagaoka (HLN) equation^46^:

2 $${\rm{\Delta }}{\rm{\sigma }}({\rm{B}})=\frac{\alpha {e}^{2}}{\pi h}[\mathrm{ln}(\frac{{B}_{\varphi }}{B})-{\rm{\Psi }}(\frac{1}{2}+\frac{{B}_{\varphi }}{B})]$$

where the prefactor α indicates the number of conducting channels, as mentioned in Eq. (1), ψ(x) is the digamma function, the dephasing length l_φ_ can be obtained from the characteristic magnetic field, ${B}_{\varphi }=\hslash /(4e{l}_{\varphi })$, e is the electronic charge, and h is Planck’s constant.

**Figure 4:**
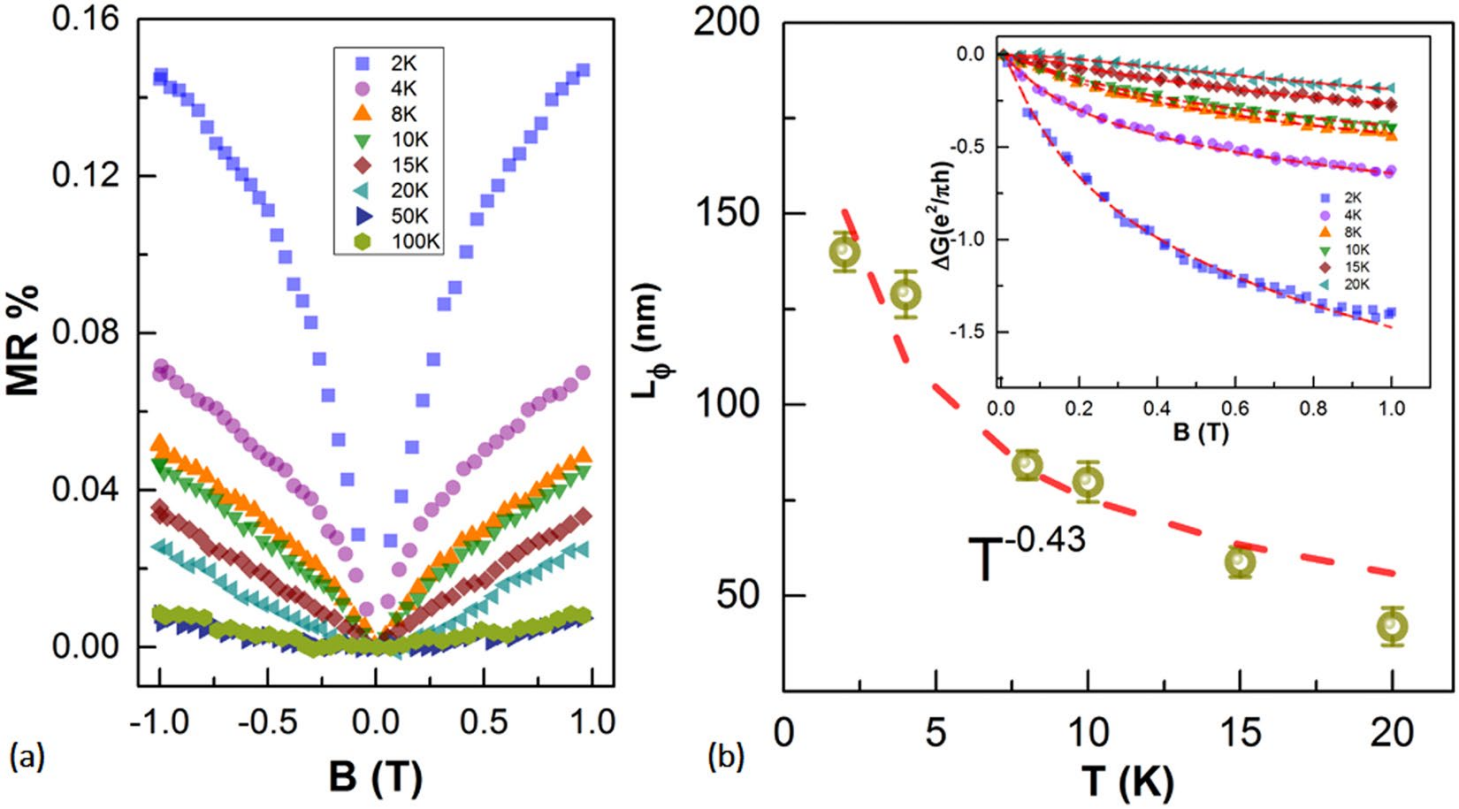
(**a**) Normalized low-field magnetoresistance ΔR/R(0T) measured in [GT_4_/ST_2_]_300 °C_ at various temperatures from 100 to 2 K, showing a pronounced WAL characteristic cusp. (**b**) Dephasing length L_φ_ as a function of temperature obtained from fitting by the 2D simplified HLN equation at low magnetic field. The dashed line in the plot shows the power-law dependence on temperature, yielding an L_φ_ decay with a T^−0.43^ dependence.

In line with work on the temperature dependence of conductance at low T, we first show the magnetoresistance (MR = ΔR/R(0 T) = (R_XX_(B) − R(0))/R_XX_(0)) of the sample [GT_4_/ST_2_]_300 °C_ in Fig. 4(a). The magnetoresistance remained positive, but the shape of the magnetoresistance changed from parabolic to a sharp resistance cusp as the temperature was decreased below 15 K, indicating the WAL effect.

As shown in the inset of Fig. 4(b), the magnetoconductance extracted from magnetoresistance was well fitted by the simplified HLN equation (1) at low temperatures and low magnetic field strength by subtracting the classical magnetoresistance contribution. This fit contained two free parameters, the coefficient α and l_φ_. Here the absolute value of α ≈ 1.206 was roughly consistent with the value derived from Eq. (1), verifying the validity of the two additional conducting channels. The dephasing length is given by ${l}_{\varphi }\propto {T}^{-0.5}\,$for 2D systems, where electron-electron scattering interferes with coherent processes^47^. A power law fit of the dephasing length l_φ_ with temperature from 15 to 2 K showed a relationship of ${l}_{\varphi }\propto {T}^{-0.43}$ fo in Fig. 4(b). The exponent was very close to 0.5, as expected for 2D or quasi-2D systems. Furthermore, the dephasing length was no more than 150 nm and much smaller than the film thickness (300 nm). This result is also consistent with the topological states found in iPCM and GeTe-Sb_2_Te_3_ multilayers^20,21^. Thus, the existence of a topological state was confirmed in our crystalline GeTe-rich superlattice system. However, considered the topological surface states can exist at interfaces of superlattice^39^, the α value around 1.2 was inconsistent with the actual superlattice period number, which contained 50 interfaces (N’ = 50), implying a strong coherent coupling of the topological surface states. The physical mechanism underlying the discordant behavior between the observed number and the predicted one is related to the quantum interference of electrons from different surface or bulk states. This process can be explained by a transport model consisting of competition between two transport parameters^32^: in-plane dephasing time τ_φ_ and interlayer tunneling τ_t_. When the condition τ_φ_ > τ_t_ is fulfilled, electrons will remain coherent before being scattered to another layer. In this case, the conducting channels are strongly coupled and the value of α is close to 0.5. Unlike the 2D vdW bond, which hinders movement of electrons, dangling bonds might increase the scattering and facilitate the tunneling^37,38^ to other sublayer blocks and give rise to the coupling of conducting channels. This result also suggests a change of the interlayer interaction and will be addressed in the following discussion.

### Dangling bond-tuned topological surfaces states coherent coupling at interfaces

**Figure 5:**
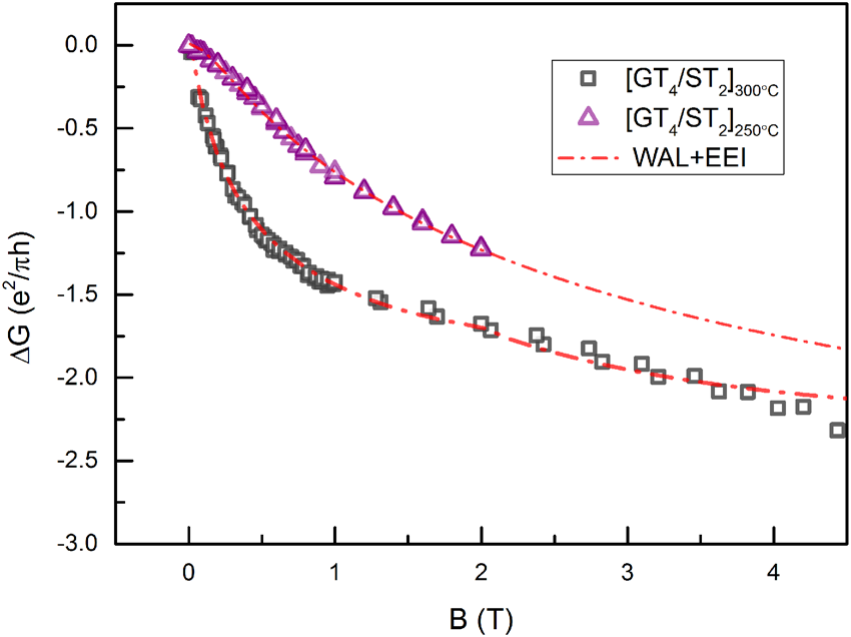
Magnetoconductance Δσ(B) of [GT_4_/ST_2_]_300 °C_ and [GT_4_/ST_2_]_250 °C_ with the perpendicular magnetic field scanning from −5 to 5 T at 2 K. Good fitting (red dashed line) to the experimental data was achieved by combining the WAL and EEI effects.

To further verify the effect of chemical bonds mentioned above on the topological surface states, we prepared another group specimens [GT/ST]_250 °C_ for comparison. These samples shared the same block thickness and electrode fabrication, which help to exclude other factors that might contribute to partially decoupled interfacial states^39^. Known from an earlier work^15^, annealing at high temperature may increase the number of dangling bonds via atomic diffusion and intermixture to achieve a lower interfacial energy. And this results is also supported by our XPS studies. Figure 5 plots the full range of magnetoconductance curves for both samples from −5 to 5T. Both the magnetoconductance curves were well fitted by the original HLN equation^46^ combined with the EEI effect. More details of the fitting equations and processing are referred to in the supplementary material. The error bar in Table 2 is determined by several measurements at different temperatures.

**Table 2:** Measured and calculated transport parameters of GeTe-rich GeTe/Sb_2_Te_3_ superlattices annealed at 250 and 300 °C for comparison. The electric conductivity σ, carrier concentration n, Hall mobility μ, and the characteristic lengths l_φ_, L_e_ and l_so_ were calculated at 2 K.

Parameters	[GT_4_/ST_2_]_250 °C_	Comparison	[GT_4_/ST_2_]_300 °C_
Thickness (nm)	300	=	300
σ_ab_ (S cm^−1^)	98.4	<	408
ρ_c_ (Ω cm)	37.6	>	19.4
n_Hall_ (10^21^ cm^−3^)	0.87	<	3.2
μ (cm^2^ V^−1^s^−1^)	0.76	≈	0.8
α (WAL)	1.6 ± 0.2	>	1.209 ± 0.06
le (nm)	3.2 ± 0.3	≈	4.1 ± 0.2
l_φ_ (nm)	41.3 ± 7	<	128 ± 4
l_so_ (nm)	6.5 ± 0.5	<	11.9 ± 0.3

Besides of the XPS studies, an increase of the dangling bonds upon Joule heating was also suggested by the change of carrier density. Carrier density, which is dominated by the vacancy concentration in Ge-Sb-Te alloy^48^, characterizes the electron interaction property. The number of vacancies at interfaces is increased as dangling bonds form. Furthermore, the ratio between the spin-orbit length l_so_ and elastic length l_e_ of [GT/ST]_300°C_ was different from that of [GT/ST]_250 °C_. This result suggests that the spin-orbit flip derives from random scattering characterized by l_e_ and is facilitated by the inner polarized field originating from the asymmetric interfacial structure of the superlattice^49,50^. Referring to our proposed structural models, the dangling bond of GeTe has the potential to make the structural symmetry broken at interfaces, resulting in the internal electric field due to the unpaired electron. Remarkably, comparing the out-of-plane resistivity between two specimens, tunneling transport was clearly enhanced after annealing, supporting the assumption that dangling bonds facilitate the tunneling transport. Thus according to the transport model which helps explain the coherent coupling of topological surfaces states, the more dangling bonds may make the coupling stronger.

Just as we predict, upon annealing, the coupling of topological surfaces states is stronger indicated by the a smaller α value. Shown in Table 2, the value of α increased to ≈1.6 at lower annealing temperature, indicating that the topological surfaces states is less coherent coupled in [GT/ST]_250 °C_. In other words, more topological surface become coupled as the number of interlayer dangling bonds increases.

## Conclusions

In summary, we have investigated the interfacial bonding of crystalline GeTe-rich superlattices by low-temperature transport measurements. Our experimental findings agreed well with certain structures proposed by ab initio calculations. In addition to the universally identified 2D vdW bonding, the formation of dangling bonds attributed to the partial intermixing from Joule heating effects was resolved by XPS studies and analysis of topological surface states. Intriguingly, the nontrivial interfacial states are quite robust in our superlattice system and prefer to be tightly coupled rather than disappear at an applied excitation lower than the transition point. This finding is significant to figure out switching mechanism involving atomic movement and intermixing issue. Our investigations of the robust and nontrivial nature of these interfacial states will contribute to a better understanding of superlattice materials and guide the fabrication of memory cells with low switching power and multi-functional devices.

## Methods

### X-ray photoelectron spectroscopy

The samples were annealed with vacuum at certain temperature 250 °C and 300 °C. In order to detect Te-O bonds, the samples were further annealed in the atmospheric air at a much lower temperature to accelerate the oxidation. All the spectrum after the Shirley background were fitted with the Gaussian-Lorentzian product lineshapes using the Marquardt-Levenberg algorithm.

### Device fabrication and electrical measurements

As depicted in the inset of Fig. 2(a), for the in-plane longitudinal resistance R_XX_ and Hall resistance R_XY_ measurements, conventional Hall-bar structures of dimensions 1600 μm × 3200 μm were patterned on the surface of the film. With the use of photolithography and lift-off processing. Six Ti/TiW ohmic contacts were deposited with a thickness of approximately 20/100 nm. A cross-bar structure, consisting of bottom and top electrodes, was fabricated and is shown in the inset of Fig. 2(b) for the out-of-plane transport measurements. The crystalline superlattice was sandwiched by the two electrodes with a line width of 10 μm. The devices were measured in a physical property measurement system chamber. In-plane resistance R_XX_ and Hall measurements were performed in a standard four-terminal setup with current I_ex_ = 1–10 μA at a frequency f = 18.4 Hz. The out-of-plane resistance R_c_ was measured with an Agilent B1500A semiconductor parameter analyzer, with the excitation and sensor integrated in the same terminal and a small excitation current I_ex_ = 1–10 μA.

### Ab initio simulations

The ab initio calculations were performed at 0 K using the Vienna Ab initio Simulation Package (VASP) code^51^, based on density functional theory (DFT). The projector augmented wave (PAW) method^52^ with the generalized gradient approximation (GGA-PBE)^53^ for the exchange-correlation functional was used. GeTe-rich GeTe/Sb_2_Te_3_ superlattices were calculated with supercells containing 27 atoms (k-points 8 × 8 × 4). The Grimme method, which adds a van der Waals correction to the conventional Kohn–Sham DFT energy, was applied in the calculations.

## Electronic supplementary material

Supplementary Material
