# Supplementary material for: Manipulation of dangling bonds of interfacial states coupled in GeTe-rich GeTe/Sb2Te3 superlattices
Source: Sci Rep. 2017 Dec 11;7:17353. doi: 10.1038/s41598-017-17671-w (PMC5725461; doi:10.1038/s41598-017-17671-w)
Supplement: Supplementary file 1 — Supplementary Material [file 41598_2017_17671_MOESM1_ESM.pdf]

Supplementary Material For

Manipulation of dangling bonds of interfacial states coupled in GeTe-rich GeTe/Sb_2Te_3 superlattices

Zhe Yang, [1,2] *Ming Xu,* [1,2] *Xiaomin Cheng,* [1,2] *Hao Tong,* [1,2,*] *and Xiangshui Miao* [1,2,3]

[1]Wuhan National Laboratory for Optoelectronics (WNLO), Huazhong University of Science and Technology (HUST), Wuhan 430074, China.

[2]School of Optical and Electronic Information, Huazhong University of Science and Technology, Wuhan 430074, China.

[3]Wuhan National High Magnetic Field Center, Huazhong University of Science and Technology, Wuhan 430074, China.

*Correspondence and requests for materials should be addressed to H. T. (tonghao@hust.edu.cn)

Considered that the fabrication of GeTe-rich GeTe/Sb_2Te_3 superlattice is different from the conventional process,[1,2] we have to figure out the influence of inter-grain diffuse, defects scattering at interfaces on the electrical transport. The average XRD, SEM, Raman results have been demonstrated in our previous study of thermal conductivity[3] and room temperature electrical transport.[4] The structure of as-deposited GeTe rich superlattice was then studied with the scanning transmission electron micrograph (STEM), of which an overview was shown in Figure **S1**. The Z-contrast of Ge could recognize the superlattice feature of the film regarding Sb and Te atoms with approximately equal Z. The boundaries were clearly shown, and the GeTe sub-layers were darker than Sb_2Te_3 sub-layers. The thickness of each block was obtained, and the correct sputtering parameters were determined to design the GeTe rich CSL with the expecting film thickness in the manuscript.

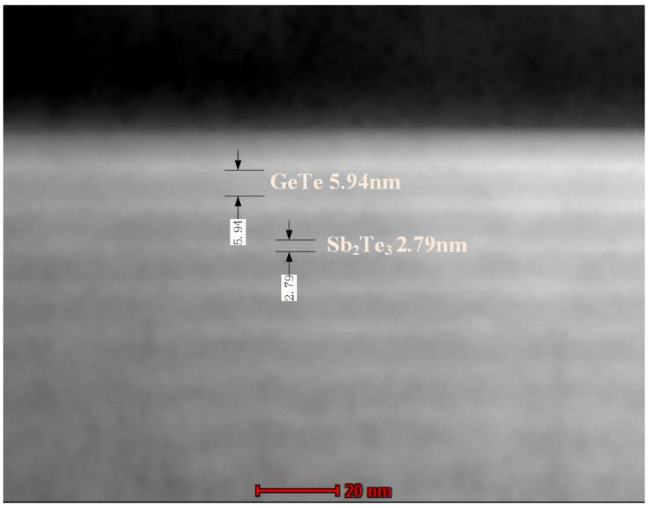

Figure S1 STEM of the as-deposited GeTe-rich GeTe/Sb_2Te_3 superlattice

(1) The grain size effect

As the annealing temperature was 250 ℃ and 300 ℃ respectively, the Joule heating

facilitated the evolution of crystalline orientation showing the merging of 20-25nm crystal grains.[4] The grains might cover the visible layer boundary, and we could not figure out the interfaces directly. Considered the size of grains and their comparison with the calculated dephasing length (around 100nm), quantum correction stemming from the inter-grain transport were likely to affect the quantum correction at low temperature.[5] Therefore, the grain effect should be discussed additionally in the following part by comparison with the in-plane transport of GeTe samples which owns a roughly same grain size.

Firstly, the grain size of both CSL and GeTe samples annealed above 250 ℃ had increased to be the approximately the same which was around 20-25nm, based on the XRD and SEM results of our previous work.[4] If we assumed that the quantum correction mainly came from the inter-grain transport, the quantum correction of GeTe was supposed to be two-dimensional (2D) and logarithmic temperature dependence which is similar to the CSL results.[6] However, just as shown in the Figure **S2**, the low-temperature (low-T) resistance exhibited an upturn and fitted better with the $T^{1/2}$ other than ln(T) temperature dependence. This fitting result meant the bulk property of the GeTe sample. In additional, the sign of the temperature dependence of quantum correction of GeTe $\Delta G/\Delta T$ was opposite to that of CSL with similar granular sizes verified by the SEM measurements. Thus the quantum correction at low T was dominated by different mechanisms other than the inter-grain scatterings. Besides of the distinct in-plane low-T transport results, we also investigated the out-of-plane temperature dependence of resistivity. For CSL specimens, as shown in the Figure1, the

out of plane transport was not proportional to the in-plane transport of CSL specimen. That was to say, in the same sample, temperature dependence of in-plane resistivity was metallic-like while that of out-of-plane resistivity was insulating-like. Thus the layered properties were prominent, and the grain boundary effect became weak in the granular superlattice structure. In an all, the scatterings from the grain boundaries were not dominant on the electronic transport of superlattice annealed at elevated temperature.

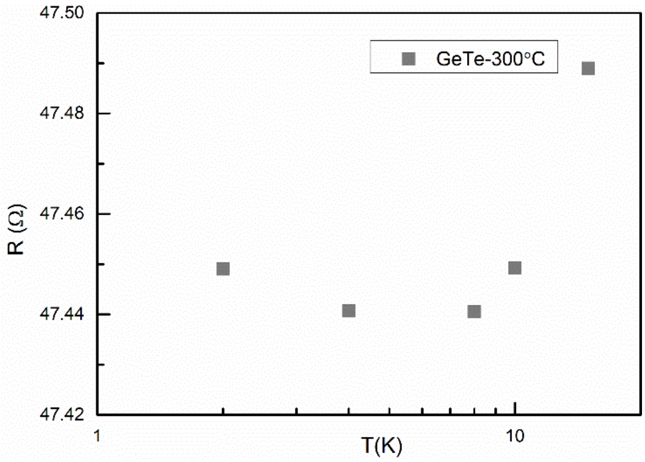

Figure S2. The temperature dependence of resistance of GeTe annealed at 300 ℃ which owned the similar grain size of CSL annealed at the same temperature condition. An upturn of resistance at low temperature was obviously shown.

(2) The fitting process of temperature dependence of conductance at low T

For the linear fitting of conductance in the form of ln (T) by WAL and EEI effects in CSL specimens, the proper fitting range was harder to determine for the whole range monotonous temperature dependence than a clear upturn in Ge-Sb-Te[6]or GeTe. This issue would be solved by the temperature dependence of the conductance applied with the gradually increased magnetic field (B-field). Just as shown in the Figure S3, monotonous behavior was broken, and an upturned tail was observed with 1T B-field

at low-T. Thus the temperature fitting range was below the upturned point of conductance correction which was about 5K. Then we introduced a new parameter κ in the unit of which was the slope of ln(T) dependence of relative conductance Δσ at low T in the unit of $h^2/\pi h$. With high B-field, the WAL effect was suppressed and so as its ln(T) dependence of Δσ. Thus the slope κ at the upper B-field was mainly dominated by quantum correction coming from the EEI effect which could be expressed:

$$\Delta\sigma_{EEI} = \frac{e^2}{2\pi^2\hbar}\left(1-\frac{3}{4}F\right)\ln\left(\frac{T}{T_0}\right) \quad (1)$$

where F is a 2D effective screening parameter. As shown in Fig. 2(d), κ value at the higher B-field was around 0.39. Thus, by using Eq(1), the F value obtained from equation (2) is about 0.81, being within the general range from 0 to 1. Meanwhile, the κ value at the lower B-field was the slope of joint ln(T) dependence of Δσ including both WAL and EEI effects. The obtained slope κ value with the various magnetic field was self-consistent and verified the choice of fitting range.

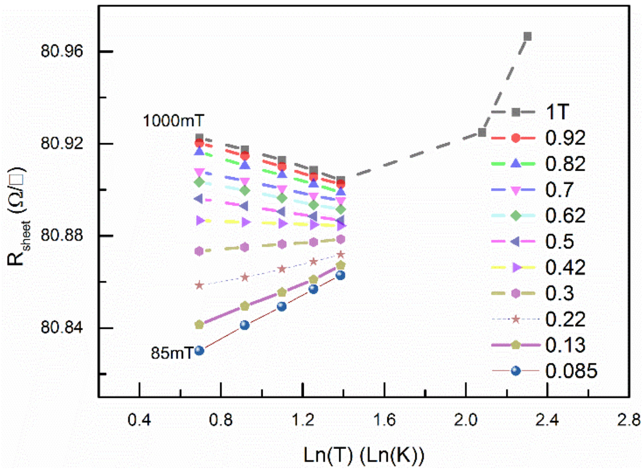

Figure S3 Sheet resistance vs. temperature for CSL samples annealed at 300 ℃, along with linear fits in ln(T). The sheet resistance of CSL samples was applied to magnetic fields from 85mT to 1T. The appropriate range was chosen from 2K to 4K which

contains 5 points for each line.

(3) The fitting process of full-range magnetoresistance

Besides of the WAL effect on the temperature dependence of conductance at low-T, magnetoresistance (MR) shows the influence of the WAL effect as well. To gain a better understanding of our MR experimental results with strong spin-orbit scattering, a full range MR from -5T to 5T B-field measurements were conducted on two sets of samples annealed at 250 ℃ and 300 ℃ respectively. The original HLN equation could not be used to fit MC well alone, which expressed as:[6,7,8]

$$\sigma_{WAL}\left(B\right)=-\frac{\alpha e^2}{2\pi^2\hbar}\left[\psi\left(\frac{1}{2}+\frac{B_e}{B}\right)-\frac{3}{2}\psi\left(\frac{1}{2}+\frac{B_\Phi+\frac{3}{4}B_{so}}{B}\right)+\frac{1}{2}\psi\left(\frac{1}{2}+\frac{B_\Phi}{B}\right)\right] \quad (2)$$

where $B_x=\frac{\hbar}{4eL_x^2}$ is the characteristic field corresponding to spin orbit (so), elastic (e), and dephasing (φ) process length scales L_x. Firstly, we used the simplified HLN equation to estimate the prefactor α and dephasing length. Then, in order to gain a more accurate fitting result, we fixed the above variable parameters and adjusted the other two spin-orbit scattering length and elastic length to fit the experimental curve. However, the fitting line deviated more severely with B-field increasing of sample $[GT_4/ST_2]_{300\,℃}$. Referred to the previous investigations,[9] the contribution from EEI effect should be taken into account which is suppressed by SOC at low B-field, written as:[10]

$$\sigma_{EEI}\left(B\right)=\begin{cases}-\frac{e^2}{2\pi^2\hbar}0.084\frac{\tilde{F}_\sigma}{2}\left(\frac{g\mu_B H}{k_B T}\right)^2\left(\frac{g\mu_B H}{k_B T}\ll 1\right)\\ -\frac{e^2}{2\pi^2\hbar}\frac{\tilde{F}_\sigma}{2}\ln\left(\frac{g\mu_B H}{1.3k_B T}\right)\left(\frac{g\mu_B H}{k_B T}\gg 1\right)\end{cases} \quad (3)$$

where g is the Lande factor, and μ_B is the Bohr magneton.[10] We applied both equations (4) and (5) to the MC data and obtained better fitting results.

(4) Hall effect

Interfaces may introduce more defects and alter the carrier density related with the band structure which was still unknown. For this purpose, Hall measurements with decreasing temperature were conducted on the CSL samples upon annealing as well as the pure sub-components GeTe and Sb_2Te_3 films. To reduce the influence of longitudinal resistance, a five-point Hall Effect geometry with a bridge was prepared to balance contribution from the diagonal term. As shown in the figure S4, the carrier densities of GeTe and Sb_2Te_3 film annealed at 300 ℃ were holes and estimated on the order of $10^{21}cm^{-3}$. These experimental results were comparable to values published in for bulk GeTe[11] and Sb_2Te_3[12], providing a high accuracy of the measuring process and preparing for the Hall measurements of the CSL samples. Meanwhile, applying the same designed Hall-bar structure, the CSL samples annealed at different temperatures exhibited the p-type property. The carrier density increased as the annealing temperature increasing. Referred to the band structure of GeTe or Sb_2Te_3, the Fermi energy of CSL is supposed to locate in the valence band as the carrier density is temperature independence. Besides, the corresponding Hall mobility μ could be roughly deduced $\mu \sim \sigma/n_e q$ by the relationship between the carrier density n_e and conductivity σ.

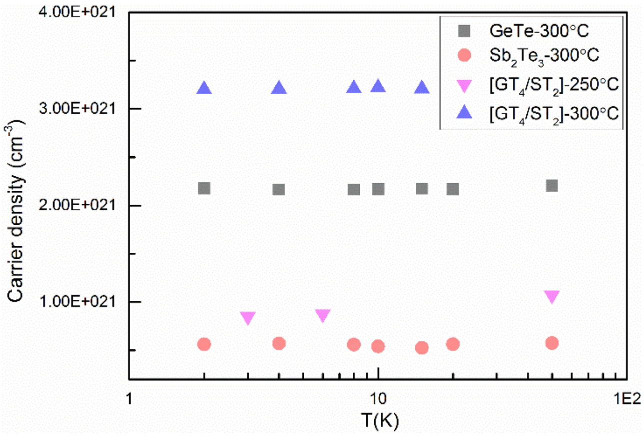

Figure S4 Temperature dependence of the carrier density measured from 2K to 50K. Both GeTe and Sb_2Te_3 films characterized by the symbol square and circle were annealed at 300 ℃ with the film thickness 150nm.

(5) **Details of the out-of-plane transport measurement setup**

Just as displayed in the Figure S5, cross-bar structure is a typical two-terminal device of which the size is clearly shown. Due to the two-terminal characteristics, the out-of-plane resistance could not obtain directly compared to the 4-terminal Hall-bar structure. First of all, we should determine the excitation range to make sure the ohmic contact by the IV measurement within Agilent B1500A semiconductor parameter analyzer.

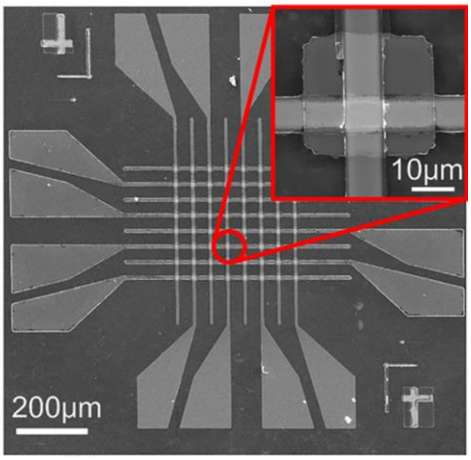

Figure S5 Scanning electron microscope images of the devices.

Seen in Figure S6, the linear IV curves provide us the available excitation range from 1-10μA. Then we fix the excitation current and obtain the total out-of-plane resistance R_c.

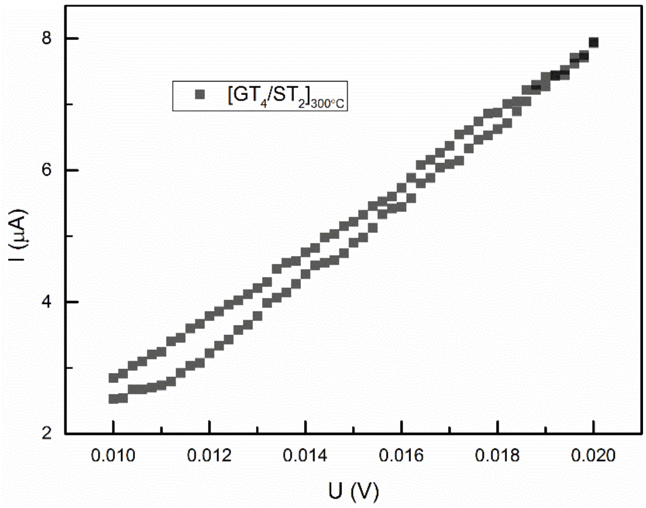

Figure S6 The linear IV curve of crossbar structure.

Besides, we also consider the component of TiW electrode resistance by $R_{TiW}=\rho_{TiW}*(L_{TiW}/10^{-5})\approx 200\ \Omega$. Meanwhile, the ohmic contact resistance can be expressed as $R_{ohm\text{-}c}=\rho_{TiW}*S_{ohmic\text{-}SL}\approx 0.1\ \Omega$. Both TiW resistivity and contact resistivity are extracted from our present work which is now in preparation. Meanwhile, both of the temperature dependence of TiW and ohmic resistance show typical metallic behavior. Thus the insulating temperature dependence behavior is the intrinsic behavior of the SL samples.

Reference

1. Tominaga, J.; Kolobov, A. V.; Fons, P. J.; Wang, X.; Saito, Y.; Nakano, T.; Hase, M.; Murakami, S.; Herfort, J.; Takagaki, Y., Giant multiferroic effects in topological GeTe-Sb2Te3 superlattices. *Sci. Technol. Adv. Mater.* **2015,** *16* (1), 014402.
2. Wang, R.; Bragaglia, V.; Boschker, J. E.; Calarco, R., Intermixing during epitaxial growth of van der Waals bonded nominal GeTe/Sb2Te3 superlattices. *Crystal Growth & Design* **2016,** *16* (7), 3596-3601.

3. Tong, H.; Miao, X.; Cheng, X.; Wang, H.; Zhang, L.; Sun, J.; Tong, F.; Wang, J., Thermal conductivity of chalcogenide material with superlatticelike structure. *Appl. Phys. Lett.* **2011,** *98* (10), 101904.

4. Tong, H.; Miao, X.; Yang, Z.; Cheng, X., Insulator-metal transition in GeTe/Sb2Te3 multilayer induced by grain growth and interface barrier. *Appl. Phys. Lett.* **2011,** *99* (21), 212105.

5. Liao, Z.-M.; Xu, J.; Zhang, X.-Z.; Yu, D.-P., The relationship between quantum transport and microstructure evolution in carbon-sheathed Pt granular metal nanowires. *Nanotechnol.* **2008,** *19* (30), 305402.

6. Breznay, N. P.; Volker, H.; Palevski, A.; Mazzarello, R.; Kapitulnik, A.; Wuttig, M., Weak antilocalization and disorder-enhanced electron interactions in annealed films of the phase-change compound GeSb 2 Te 4. *Phys.l Rev. B* **2012,** *86* (20), 205302.

7. Zhang, S.; McDonald, R.; Shekhter, A.; Bi, Z.; Li, Y.; Jia, Q.; Picraux, S. T., Magneto-resistance up to 60 Tesla in topological insulator Bi2Te3 thin films. *Appl. Phys. Lett.* **2012,** *101* (20), 202403.

8. Hikami, S.; Larkin, A. I.; Nagaoka, Y., Spin-orbit interaction and magnetoresistance in the two-dimensional random system. *Prog. Theor. Phys.* **1980,** *63* (2), 707-710.

9. Zhao, Y.; Liu, H.; Guo, X.; Jiang, Y.; Sun, Y.; Wang, H.; Wang, Y.; Li, H.-D.; Xie, M.-H.; Xie, X.-C., Crossover from 3D to 2D quantum transport in Bi2Se3/In2Se3 superlattices. *Nano Lett.* **2014,** *14* (9), 5244-5249.

10. Lee, P. A.; Ramakrishnan, T., Disordered electronic systems. *Rev. Mod. Phys.* **1985,** *57* (2), 287.

11. Zuev, Y. M.; Lee, J. S.; Galloy, C.; Park, H.; Kim, P., Diameter Dependence of the Transport Properties of Antimony Telluride Nanowires. *Nano Lett.* **2010,** *10* (8), 3037-3040.
